# Single-centre experience with peptide receptor radionuclide therapy for neuroendocrine tumours (NETs): results using a theranostic molecular imaging-guided approach

**DOI:** 10.1007/s00432-023-04706-1

**Published:** 2023-04-01

**Authors:** S. Gordon, D. L. H. Chan, E. J. Bernard, M. E. Eslick, K. P. Willowson, P. J. Roach, A. F. Engel, R. Maher, S. J. Clarke, V. Agarwal, L. Yasmin, M. De Silva, S. Mascall, A. Conner, D. Nevell, N. Pavlakis, D. L. Bailey

**Affiliations:** 1 Sydney Vital Translational Cancer Research Centre, Sydney, Australia; 2 Department of Medical Oncology, Royal North Shore Hospital, Sydney, Australia; 3 Faculty of Medicine & Health, University of Sydney, Sydney, Australia; 4 Department of Nuclear Medicine, Royal North Shore Hospital, St Leonards, Sydney, NSW 2065 Australia; 5 Department of Colorectal Surgery, Royal North Shore Hospital, Sydney, Australia; 6 Department of Medical Imaging, Royal North Shore Hospital, Sydney, Australia; 7 Bill Walsh Translational Cancer Research Laboratory, University of Sydney, Sydney, Australia; 8 Department of Anatomical Pathology, Royal North Shore Hospital, Sydney, Australia

**Keywords:** Neuroendocrine, Peptide Receptor Radionuclide Therapy (PRRT), PET, NETs, Multidisciplinary Team (MDT)

## Abstract

**Aim:**

To summarise our centre’s experience managing patients with neuroendocrine tumours (NETs) in the first 5 years after the introduction of peptide receptor radionuclide therapy (PRRT) with [^177^Lu]Lu-DOTA-octreotate (LUTATE). The report emphasises aspects of the patient management related to functional imaging and use of radionuclide therapy.

**Methods:**

We describe the criteria for treatment with LUTATE at our centre, the methodology for patient selection, and the results of an audit of clinical measures, imaging results and patient-reported outcomes. Subjects are treated initially with four cycles of ~ 8 GBq of LUTATE administered as an outpatient every 8 weeks.

**Results:**

In the first 5 years offering LUTATE, we treated 143 individuals with a variety of NETs of which approx. 70% were gastroentero-pancreatic in origin (small bowel: 42%, pancreas: 28%). Males and females were equally represented. Mean age at first treatment with LUTATE was 61 ± 13 years with range 28–87 years. The radiation dose to the organs considered most at risk, the kidneys, averaged 10.6 ± 4.0 Gy in total. Median overall survival (OS) from first receiving LUTATE was 72.5 months with a median progression-free survival (PFS) of 32.3 months. No evidence of renal toxicity was seen. The major long-term complication seen was myelodysplastic syndrome (MDS) with a 5% incidence.

**Conclusions:**

LUTATE treatment for NETs is a safe and effective treatment. Our approach relies heavily on functional and morphological imaging informing the multidisciplinary team of NET specialists to guide appropriate therapy, which we suggest has contributed to the favourable outcomes seen.

## Introduction

The management of individuals with a somatostatin receptor (SSTR) expressing neuroendocrine tumours (NETs) has changed significantly in Australia over the past decade. The introduction of peptide receptor radionuclide therapy (PRRT) with yttrium-90 (^90^Y) or lutetium-177 (^177^Lu) using a theranostic^1^ approach has demonstrated a significant impact on the management of the disease (Strosberg et al. 2017). These radionuclides emit β^−^ and γ radiation during nuclear decay that can damage the DNA of cells and, when attached to a highly targeted vector such as a molecule, peptide or antibody, are capable of delivering systemic therapy for metastatic disease. The theranostic paradigm uses the same targeting agent for both molecular imaging and therapy (Kelkar and Reineke 2011), imaging of the disease (e.g. using ^68^Ga or ^18^F with positron emission tomography (PET)) to demonstrate the degree of uptake of the radiotracer, and, if sufficient uptake is shown, to replace the imaging radionuclide with a therapeutic radionuclide and proceed to treatment. The results of the NETTER-1 trial which treated NET patients with [^177^Lu]-DOTA^0^,Tyr^3^-octreotate (LUTATHERA^®^, Novartis), demonstrated a significant improvement in both progression-free and overall survival compared to conventional therapy with somatostatin analogues (SSAs) (*1*). Progression-free survival at month + 20 after commencing treatment was 65% in the LUTATE group versus 11% in the control group. However, in spite of the impressive results, the selection of the subjects for treatment could be considered, by today’s standards, less than optimal as not all of the investigators had access to PET imaging with radiolabelled SSAs.

We hypothesised that the results of NETTER-1 could be improved with better patient selection based on:

1. establishing a dedicated NET specialist centre,
2. staffing the centre with NET-specialised medical oncologists, clinical scientists, NET-dedicated nurses and other medical specialities, and
3. relying heavily on functional imaging with PET to guide decision making.

In Australia, PET imaging with a radiolabelled SSA such as [^68^Ga]Ga-DOTA-octreotate (“DOTATATE”), often referred to as somatostatin receptor imaging (SSTRI), is often supplemented with a [^18^F]FDG PET scan (“dual-PET” imaging) to give a more complete overall assessment of the status of the disease arising from assumed co-existing different clones of the disease in the individual. Further, we have integrated the imaging results from the two PET scans into a single score known as the NETPET score which is graded from 0 to 5 (Chan et al. 2017) where a NETPET grade of 0 (P0) represents both normal DOTATATE and FDG PET scans, grade 1 (P1) represents DOTATATE positive but FDG negative disease at all sites in the body, grade 5 (P5) represents the opposite, namely, DOTATATE negative but FDG positive disease, and grades 2–4 represent the spectrum of varying positivity of uptake on both scans. In general, patients with NETPET grades of P1–P4 would potentially meet our centre’s criteria for consideration of [^177^Lu]-DOTA^0^,Tyr^3^-octreotate (“LUTATE”) therapy if the uptake of DOTATATE and FDG (if present) is predominantly spatially concordant. It is generally accepted that high FDG uptake in the absence of DOTATATE uptake (i.e. NETPET Grade P5) is a poor prognostic indicator (Binderup et al. 2010), whilst, conversely, high DOTATATE uptake (Grades P1–P3), especially in the setting of low overall burden of disease, represents a more favourable prognostic marker for using PRRT as an option (Campana et al. 2010, Tirosh et al. 2018).

In this report, we present the results of the first 5 years’ experience with LUTATE therapy at our NET Centre at Royal North Shore Hospital, Sydney, New South Wales, where we have ready access to PET imaging with both DOTATATE and FDG as well as LUTATE therapy.

## Patient recruitment, selection and management

To give some demographic context, the Australian state of New South Wales (NSW) has ~ 8.2 m residents and covers just over 800,000 km^2^, 10% of the total land mass of Australia, making it over twice the size of the nation of Germany, but with less than 10% of its population. Over 70% of NSW residents live in the extended metropolitan area of the state capital, Sydney, and the contiguous urban enclaves joining it with the cities to the immediate north (Newcastle) and south (Wollongong).

Prior to 2015, there was limited access to LUTATE therapy for NET patients in NSW, with only one centre providing limited service. In 2015, the NSW Ministry of Health provided state-wide funding for LUTATE therapy through the Cancer Institute NSW (CINSW) to be provided at two centres, Royal North Shore (RNS) Hospital in northern Sydney and St George Hospital in southern Sydney, under an evaluation framework, the *NSW Lutate Therapy Referral and Protocol for Neuroendocrine Cancer Patients* (ACI-NSW 2015). In 2019 CINSW produced a report of the combined outcomes of the two centres from 2010 to March 2017 (Lin et al. 2019).

All subjects who were treated with LUTATE in our centre were referred by either local or remote clinicians to the Northern Sydney Local Health District (NSLHD) Neuroendocrine Tumour Multi-Disciplinary Team (MDT) meeting which is held fortnightly. The meeting is attended by all clinical specialists involved in the management of NET patients including medical oncologists, surgeons, radiation oncologists, radiologists, palliative care specialists, anatomical pathologists and nuclear medicine physicians, plus the clinical nurse co-ordinator (CNC), advanced trainees in the various disciplines, basic and applied scientists, allied health professionals and medical students. The Northern Sydney NET Unit and its MDT was recently accredited as a NET *Centre of Excellence* by the European Neuro-Endocrine Tumour Society (ENETS) (ENETS 2022), joining one other accredited centre in Australia and over 50 others globally, mostly in Europe.

An essential requirement for receiving LUTATE under the *NSW Lutate Therapy Protocol* was that the subject was discussed at a NET-specific MDT meeting and that the MDT recommendation was to treat with LUTATE. The inclusion criteria for treating with LUTATE were:

- Histologically proven NET of any origin.
- Locally advanced and/or inoperable (metastatic) disease.
- Failed first-line systemic therapy.
- Progressive disease demonstrated radiologically while on SSA therapy or uncontrolled symptoms despite systemic therapy.
- Presence of somatostatin receptors on the known tumour lesions demonstrated by [^68^Ga]DOTATATE PET scan within the past 6 months. The uptake of the NET lesions should be at least as high as normal liver uptake.
- ECOG status 0–2.
- Patient written informed consent.

Over time, the MDT found it extremely useful to have more than one DOTATATE scan available if at all possible to assess the trajectory, or tempo, of the disease to assess the rate of progression on functional imaging. The role of combined DOTATATE and FDG PET imaging will be discussed later.

Exclusion criteria for LUTATE treatment included:

- poor renal function (glomerular filtration rate (GFR) < 40 mL/min),
- life expectancy < 3 months, or
- histological evidence of grade 3 neuroendocrine carcinoma (NEC).

Referral to the NET MDT did not imply transfer of primary patient management to a medical oncologist in our centre, with the referrers (especially the medical oncologist referrers) usually retaining routine management of the patient.

## Methods

### LUTATE therapy and imaging protocol

All subjects selected for PRRT were generally planned to have a course of four cycles each of 7–8 GBq of LUTATE separated at eight weekly intervals over a 6-month period. The amount of LUTATE administered was standardised without any allowance for factors such as body habitus or burden of disease present. This treatment protocol follows the commonly used approach based on the original Erasmus Medical Centre procedure (Kwekkeboom et al. 2003).

Subjects recommended for LUTATE by the NET MDT would have an extensive workup including a consultation by the nuclear medicine physician with renal imaging and measurement of radionuclide-based GFR. If these and all other investigations and assessments were acceptable, they would then cease long-acting SSA therapy at least 1 month prior to the intended treatment date. Subjects were individually considered for a radiosensitising dose of capecitabine (Xeloda^®^) 7 days prior to each treatment cycle of LUTATE (1000–1500 mg/m^2^ once daily orally) and continued for 14 days after treatment. On the day of treatment, prior to LUTATE infusion, the subjects were given an oral dose of 8 mg of a serotonin-blocking agent (ondansatron—a 5-HT_3_ agonist) to suppress nausea as well as a steroid (dexamethasone 8 mg oral) and asked to take 4 mg of both once per day for the subsequent 2 days after treatment.

LUTATE is radiolabelled on site using either “carrier-added” lutetium-177 sourced from Europe, where the ^177^Lu is manufactured in a nuclear reactor using ^176^Lu as the starting product (^176^Lu(n, β^−^) ^177^Lu), or “non-carrier-added” ^177^Lu manufactured in the OPAL Research Reactor at ANSTO, Lucas Heights, Sydney, using ^176^Yb as the starting product (^176^Yb(n, β^−^)^177^Yb⟶^177^Lu), where the intermediate product, ytterbium-177, has a short physical half-life of 1.9 h. The LUTATE radiopharmaceutical formulation is prepared up to 24 h prior to treatment and kept in a refrigerator at −33°C. Details of the production method can be found in the article by Aslani et al (2015).

On the day of treatment, all subjects received an infusion of amino acids (either locally in-house produced 25 g lysine and 25 g arginine per litre or a commercial product (Synthamin^®^, Baxter International Inc.) containing 5.8 g lysine and 11.5 g arginine per litre) for renal protection over a 4-h period commencing 30 min prior to the injection of LUTATE. The LUTATE is prepared in a volume of 18 mL and delivered using an infusion pump over 20 min, commencing at a slow infusion rate and then increasing in a stepwise, ramped manner as long as the subject does not experience any nausea or other symptoms (in order: 6 mL/h for 1 min, 20 mL/h for 5 min, 40 mL/h for 5 min, 60 mL/h for 5 min and finally 100 mL/h until completed).

We employed an intensive post-therapy imaging protocol for the first 5 years of LUTATE treatment. On the day of treatment, the subjects would initially have a whole-body anterior/posterior (A/P) whole-body planar transmission scan on the gamma camera prior to receiving the LUTATE using a cobalt-57 (^57^Co) sheet source attached to one of the detectors and low-energy collimators, for subsequent use in the estimation of the total radioactivity retained in the body over time using the MIRD approach to planar image quantification (Siegel et al. 1999). This was followed by whole-body planar imaging at the end of the LUTATE infusion but prior to any losses by voiding urine from the body plus a SPECT/CT scan of the abdomen to image the kidneys as the organs at most risk. All image data were acquired on a SPECT/CT system (Intevo.6, Siemens Healthineers, Hoffman Estates, USA) with NaI(Tl) gamma camera detector thickness of 16 mm using medium energy (MELP) collimators and a 15% energy window centred on the 208 keV photopeak. The X-ray CT data were acquired with automatic exposure control at 130 kV_p_ tube voltage and nominal 50 mAs beam current without intravenous contrast. A radionuclide standard containing approximately 40–50 MBq ^177^Lu was included in the field of view for both planar whole body and SPECT scans. These scans were repeated at 4 h, 24 h and 96–120 h post-infusion to provide sufficient time points to fit time–activity curves to monitor LUTATE biodistribution over time and calculate organ dosimetry. Full technical details can be found in the papers that we have previously published (Bailey et al. 2015a, b).

Due to the aetiology of NETs, quality of life (QoL) may be significantly impacted by tumour growth as well as associated hormonal production. From the start of treating subjects with LUTATE, we have monitored quality of life using EORTC validated surveys. The QLQ-C30 survey (Aaronson et al. 1993) is a general questionnaire containing 30 questions about the QoL for a cancer patient for five functional scales (Physical, Role, Cognitive, Emotional and Social Functioning), three symptom scales (Fatigue, Pain and Nausea/Vomiting), and a global health status/QoL scale. Six single item scales are also included (Dyspnoea, Insomnia, Appetite Loss, Constipation, Diarrhoea and Financial Difficulties). In addition, we have asked the subjects to complete the EORTC QLQ-GI.NET21 survey (Yadegarfar et al. 2013) which is a NET-specific survey of 20 questions about symptom frequency and the impact these have on their lifestyle. The subjects were asked to complete the survey whenever they attended the Department of Nuclear Medicine and all responses are stored in a NET-specific REDCap database (Harris et al. 2009, 2019). All subjects in this report gave written informed consent to their data being used for the purposes of audit, training, education, research and review.

### Clinical audit

A clinical audit was conducted on all NET patients treated with LUTATE at the Royal North Shore Hospital between August 2013 and December 2018. This was a case note review of medical records available to the Departments of Medical Oncology and Nuclear Medicine. Data in the medical records were recorded primarily for clinical care and not specifically for the purpose of retrospective analysis. Clinicopathological and outcome data were extracted from chart review after local Human Research Ethics Committee (HRE)C approval. All subjects gave written informed consent prior to being treated as well as allowing their data to be used for audit and research purposes.

One outcome for this audit was to assess overall survival (OS) which was defined from the decision to offer PRRT treatment to death or last follow-up. Secondary outcomes were progression-free survival (PFS) and radiographic response by investigator assessment according to RECIST V1.1. PFS was measured from the date of first PRRT treatment to evidence of radiological progression (or death, or last follow-up as relevant).

### Impact of LUTATE on renal and bone marrow function

Prior to the first cycle of LUTATE, [^99m^Tc]Tc-DTPA renal dynamic imaging and blood samples were taken to assess kidney function with radionuclide-based GFR plus full blood count (FBC), liver function tests (LFTs), electrolytes, urea and creatinine (EUC) and hence estimated renal GFR (eGFR). Measured GFR is tested mid-treatment between Cycles 2 and 3, and then ~3 months after the final LUTATE cycle. Blood tests were performed 2 weeks prior to each cycle where another sample is taken for EUC, LFTs, FBC and serum Chromogranin A.

### Renal and bone marrow dosimetry

The biokinetics of LUTATE demonstrate rapid blood clearance and urinary excretion (Bailey et al. 2015a, b) and as such the organs at risk are considered to be bone marrow and kidneys. The dose limiting organ is usually the kidney, due to the active resorption of LUTATE (or any somatostatin labelled radionuclide) within the proximal tubule. Subjects who had completed all four cycles of LUTATE with a minimum of imaging at 4, 24 and 96–120 h post-injection at each cycle were considered for dosimetry review (Willowson et al. 2018). From that subset of patients, those that had renal function measurements (GFR) at baseline and follow-up measurements at least 3 months after the last cycle were considered for renal review. Imaging for renal dosimetry consisted of quantitative SPECT/CT (Willowson et al. 2008) with the kidneys positioned in the centre of the axial field view. Image quantification at each time point involved CT-derived correction for scatter and attenuation, as well as application for a camera-specific dead time correction (performed on each projection) and sensitivity factor.

Renal dose estimates were derived by defining kidney cortical volumes of interest (VOIs) on the coregistered CT data and referencing the calculated percent injected activity in the organs at each time point to fit time–activity curves in a radiation dose estimation software package, OLINDA-EXM (Stabin et al. 2005). The appropriate adult model (male or female) was chosen with correction for patient-specific kidney mass derived from the segmented CT. The data were scaled by the net injected radioactivity for each cycle to arrive at total absorbed renal dose (in units of Gray [Gy]) using all imaging data.

The dosimetry estimate for the bone marrow was adapted from the method of Stabin (2008) based on the model originally proposed by Cristy (1981). Volumes of interest were defined on the fourth and fifth lumbar vertebra (L4 and L5) on the CT scan and transferred to the SPECT scan. The Cristy model assumes that the bone marrow in L4 and L5 together accounts for ~ 7% of total bone marrow. From these measurements an estimate for total bone marrow radiation dose can be made.

### Quality of life assessments

Subjects were asked to fill out the questionnaires prior to their first LUTATE cycle, at each visit prior to their subsequent cycles, and at least 3 months after receiving their final cycle of LUTATE**.** The primary analysis involved comparing changes from baseline to post-LUTATE and a sub-analysis involved assessing midcycle changes of QoL. The inclusion criteria for including the QoL assessments in our analysis were:

- completed all four cycles of LUTATE treatment,
- ECOG status 0–2 (Oken et al. 1982),
- completed QLQ-C30 survey prior to the first cycle,
-  > 50% of questions were completed at every visit, and
- QLQ-C30 survey completed at least 3 months after the last LUTATE cycle.

### Statistical analysis

Descriptive statistics were used to evaluate the study population with the Kaplan–Meier method to evaluate OS and PFS.

#### Renal function

To assess the impact of LUTATE treatment on renal function, a Wilcoxon matched-pairs signed-ranks test was used to compare the GFR at baseline with the value at the 3 months post-Cycle 4 follow-up as the data did not pass an initial normality test. Two-way ANOVA with Dunnett’s multiple comparison test was performed to assess difference in patients that received less than a total 10 Gy renal dose compared to patients who received over 10 Gy total renal dose. Statistical significance was considered at the level of *p* < 0.05. We also examined whether the kidney dosimetry for Cycle 1 was correlated with GFR, as it might be expected that subjects with poor renal function and hence slower transit of the radiotherapeutic might receive higher radiation doses to the kidneys which could become a contraindication for further treatments with LUTATE.

#### QoL comparisons

For primary and sub-analysis of QoL, a two-way ANOVA with Dunnett’s multiple comparison test was performed. Statistical significance was considered at p < 0.05. GraphPad Prism (Version 8.4.3) software was used for all QoL-related statistical analyses.

## Results

### Subject characteristics

In the period from August 2013 and December 2018, a total of 418 individual subjects were discussed at the NET MDT meeting of which 143 were referred for LUTATE therapy. Subject and clinical characteristics of those treated with LUTATE are presented in Table 1*.* Median age at the time of first LUTATE cycle was 65 years (range 20–87 years)*.* The majority of subjects were from metropolitan areas (57%) with the remainder from rural NSW and one from overseas.

**Table 1:** Demographic and baseline clinical characteristics of all patients who underwent LUTATE treatment at Northern Sydney NET Unit during the audit period 2013–2018

Characteristic	No. of subjects
Gender	
Male	69 (48%)
Female	73 (52%)
Age at first LUTATE treatment (years)	
Mean	61 ± 13
Median	65
Range	20–89
Primary site of tumour/disease	
Small bowel	59 (42%)
Pancreas	41 (28%)
“Other histology NET”	13 (9%)
Bronchial	8 (6%)
SSTR positive non-NET malignancy	8 (6%)
Unknown	5 (4%)
Colorectal	3 (2%)
Other	5 (4%)
Total	142
Reason for treatment (CINSW criteria)	
Disease progression	100 (70%)
Uncontrolled symptoms	10 (8%)
Both	32 (22%)
Grade (WHO criteria)	
Grade 1	41 (29%)
Grade 2	49 (35%)
Grade 3	16 (11%)
Unknown/unable to be determined	35 (25%)
Prior treatment	
Somatostatin analogues (long or short acting)	108 (76%)
Surgery	88 (62%)
Chemotherapy	37 (26%)
Liver-directed therapy	19 (13%)
External beam radiotherapy	29 (20%)
Sites of metastases at time of LUTATE treatment	
Liver	112
Lymph nodes	97
Liver + lymph nodes	82
Skeleton	70
Liver + skeleton	55
Extensive metastatic disease	43

### Reasons for recommending LUTATE

The majority (92%) of subjects (132 out of 142) were recommended for LUTATE based on progressive disease. Of these, 32 subjects also exhibited uncontrolled carcinoid symptoms. Only 10 (7%) subjects were treated with LUTATE based primarily on the associated elevated hormonal levels and the accompanying carcinoid symptoms alone. The most common carcinoid symptoms were flushing, diarrhoea, uncontrolled weight gain or loss, chronic nausea and vomiting.

### Treatments administered

During August 2013–December 2018, 537 cycles of LUTATE were administered at our centre. The introduction of state government funding for LUTATE therapy by the NSW Ministry of Health in 2015 led to an increase in both number of cycles and new subjects. The number of cycles roughly doubled from 28 in 2014 to 58 in 2015 and subsequently again doubled to 126 cycles in 2016. The mean injected amount administered over the audit period was 7761 ± 309 MBq, with a median of 25 weeks between the first and fourth cycle (range 18–45). At the time of the censor, 15 subjects had not yet completed their full four cycles of LUTATE but later went on to finish their treatment in the first part of 2019. Twenty-three subjects were unable to finish all four cycles of LUTATE. The most common reasons for LUTATE treatment withdrawal were rapid response to treatment (i.e. no targetable disease remaining), low platelet levels or deceased.

### Re-treating with LUTATE

Seventeen subjects (11%) treated during the audit period went on to receive further cycles of LUTATE beyond their initial four cycles, with three subjects receiving ten cycles of LUTATE in total. During the audit period, 13 subjects received re-treatment after the standard four cycles.

The median interval between subsequent rounds of LUTATE treatment was 21 months (range 15–68 m). The main reason for re-treating was progressive disease as demonstrated clinically and/or by increased tumour avidity on DOTATATE PET scans and/or new sites of disease appearing. One subject was re-treated with LUTATE based on successful prior symptom control and pain management.

### Overall survival and progression-free survival

**Fig. 1:**
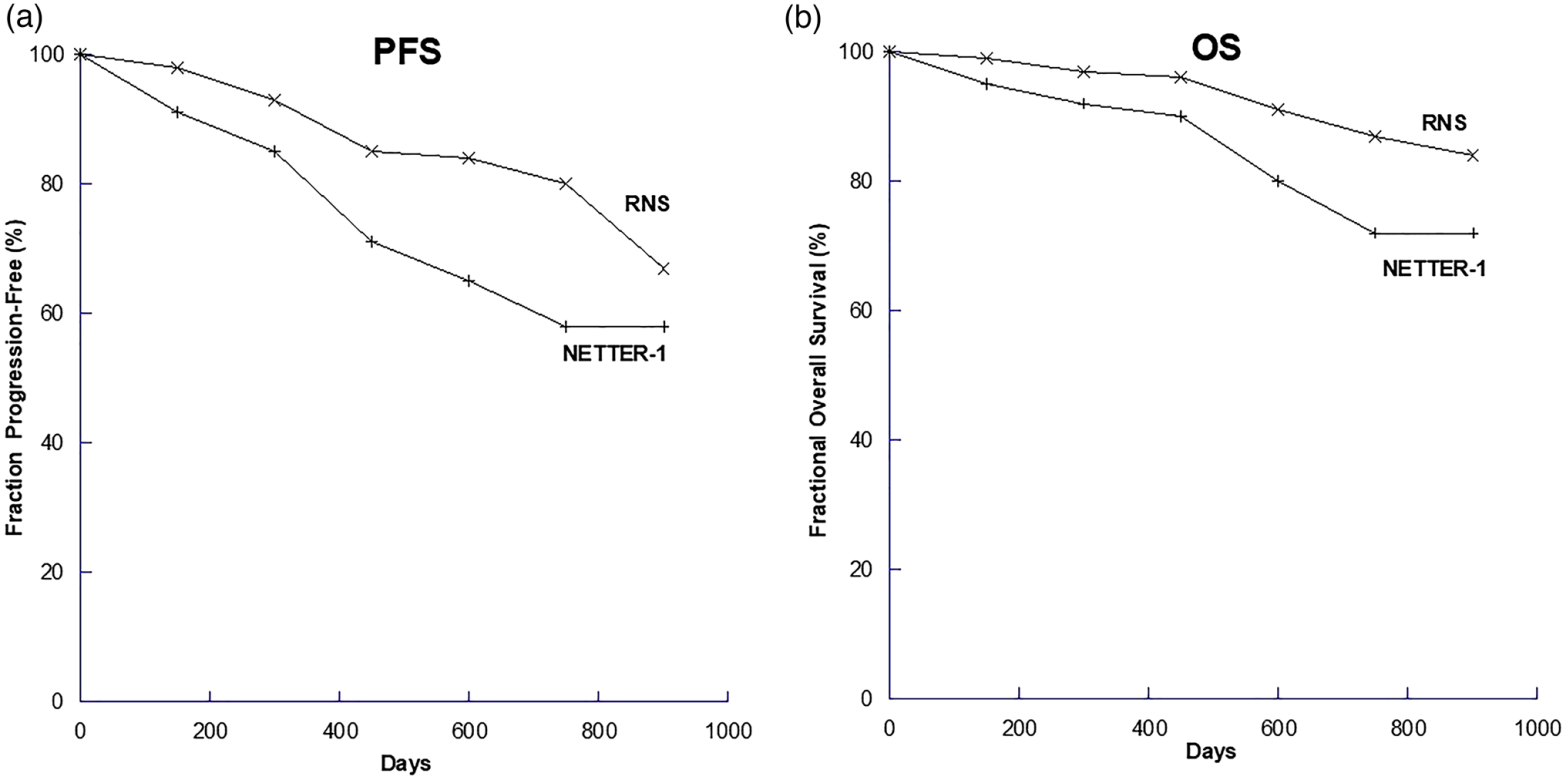
Kaplan–Meier curves of **a** progression-free survival (PFS) and **b** overall survival (OS) from the NETTER-1 trial and our NET centre at RNS. The NETTER-1 trial started to count from randomisation onto the trial, while our starting point was the decision to offer LUTATE treatment

From commencing our comprehensive NET service and using all imaging and clinical indications in our cohort we have found a median progression-free survival interval of 34.3 months and a median overall survival of 72.5 months (Fig. 1). These values compare favourably with the results of the NETTER-1 trial (1).

### Renal and bone marrow dosimetry

The average total renal dose in our cohort for all four cycles of LUTATE was 10.6 ± 4.0 Gy (range: 3.5–27.6 Gy). The mean radiation dose to kidney normalised by administered radioactivity per cycle was 0.36 ± 0.16 Gy/GBq (range: 0.04–1.13 Gy/GBq) [*N* = 333]. The average total bone marrow dose for all 4 cycles of LUTATE was 0.12 ± 0.04 Gy (range 0.09–0.18 Gy). The mean radiation dose to bone marrow was 3.8 ± 3.6 × 10^−3^ Gy/GBq (range: 0.15 × 10^−3^ to 19.6 × 10^−3^ Gy/GBq) [*N* = 65].

### Renal and bone marrow function

#### Renal function

Overall, renal function was found to decrease slightly from an average baseline of 79.0 ± 20.3 mL/min/1.73 m^2^ (range 40–138) to 75.4 ± 19.8 mL/min/1.73 m^2^ (range 39–133) (*N* = 116), a decrease of ~ 5%. While this was a statistically significant decrease using a two-tailed paired *t* test (*p* = 0.0007), it was not considered to be a significant *clinical* decline in kidney function. The average follow-up period for the renal assessment from final (i.e., 4th cycle) LUTATE treatment to GFR measurement was 2.4 ± 1.9 months. When categorised into two groups of those receiving ≤ 10 Gy and > 10 Gy total renal dose, a two-way ANOVA showed no statistical difference between the two groups suggesting that the decline was not due to a higher estimated radiation dose.

Thirty-nine subjects exhibited what was considered a *clinically* significant decline in renal function, defined as ≥ 10% decrease in GFR from baseline to 3 months post-LUTATE. The renal dose estimate in these subjects showed an average total renal dose of 10.8 ± 3.1 Gy (range 6.2–18.4; *N* = 15) while subjects with stable or improved post-LUTATE renal function had an average total dose of 10.7 ± 4.9 Gy (range 3.5–27.6 Gy; *N* = 39), again indicating that a difference in renal radiation exposure between the two groups did not account for the impact on renal function. The subjects with the clinically significant decline in renal function had an average GFR at baseline of 82.9 ± 20.2 mL/min/1.73 m^2^ (50–138 mL/min/1.73 m^2^; *N* = 39) and the subjects with stable or improved renal function after completing LUTATE had an average pre-treatment baseline GFR of 77.1 ± 20.2 mL/min/1.73 m^2^ (40–137 mL/min/1.73 m^2^; *N* = 78). Finally, no correlation was seen between the baseline pre-treatment GFR and radiation dose to kidneys.

#### LUTATE treatment and bone marrow response

**Fig. 2:**
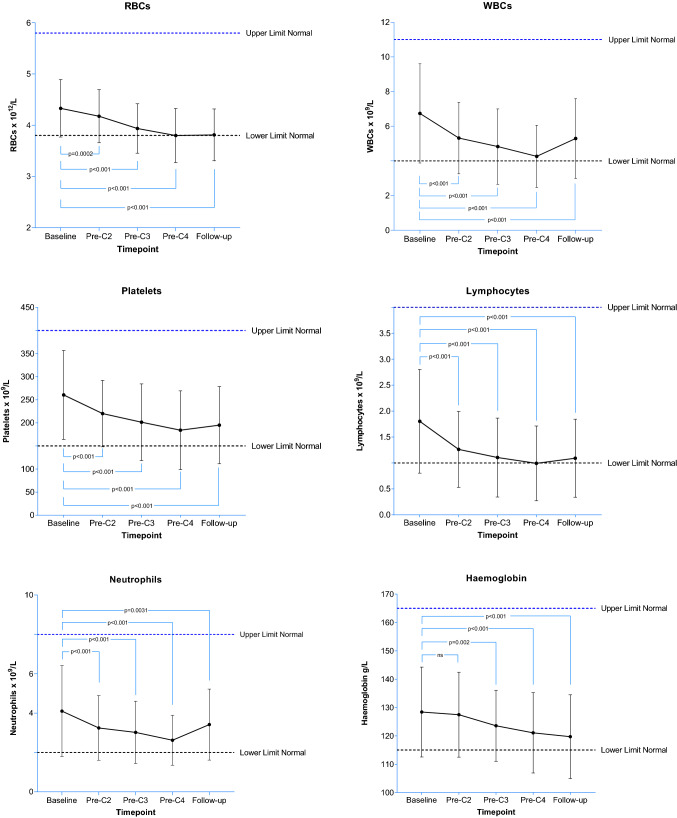
The average of the pooled subjects’ blood results prior to each cycle of LUTATE are shown plus a follow-up time point after the last cycle. The *p* values are calculated from a repeated measures ANOVA comparing each measurement to the pre-Cycle 1 baseline value. Error bars represent ± 1σ. NS—not significant (*p* > 0.05). There are 86 complete datasets in this analysis from the cohort of 142 subjects treated in the period under review

As part of the routine work-up prior to treatment a full blood count was performed before each cycle of LUTATE. In general, a decline was seen over the course of treatment in red and white blood cells, particularly neutrophils and lymphocytes (both ~ 40% decline), and platelets (Fig. 2). Most blood counts showed evidence of “rebounding” towards more normal levels after the last cycle of LUTATE. Seven of our cohort of 142 patients (5%) treated with LUTATE developed myelodysplastic syndrome which was confirmed by either bone marrow biopsy or suspected based on FBC and blood film.

### Quality of life

Evaluation of QoL for all treated subjects involved primary overall and interval assessment of EORTC survey scores. Primary analysis (*N* = 69) showed stable functional and symptom status following LUTATE treatment. Further interval assessment analysis (*N* = 48) revealed little to no change in QoL parameters during the course of LUTATE treatment. Analysis of Global Function showed no statistically significant difference between baseline (68.4 ± 19.0) and post-LUTATE (67.5 ± 21.3) (*p* > 0.05). Similar results were seen for other aggregate scores when all subjects were combined. However, differences were seen when the scores were separated by WHO grade of disease at time of treatment. Subjects with grade 1–2 disease did not display changes and remained stable over the course of treatment while subjects with grade 3 disease showed definite improvements in Global Function, Emotional Function, Physical Function and Symptom Score, while Social Function, Cognitive Function and Fatigue remained stable.

### Case reports

We include a pair of cases demonstrating the wide variability in neuroendocrine neoplasia that we have encountered.

**Fig. 3:**
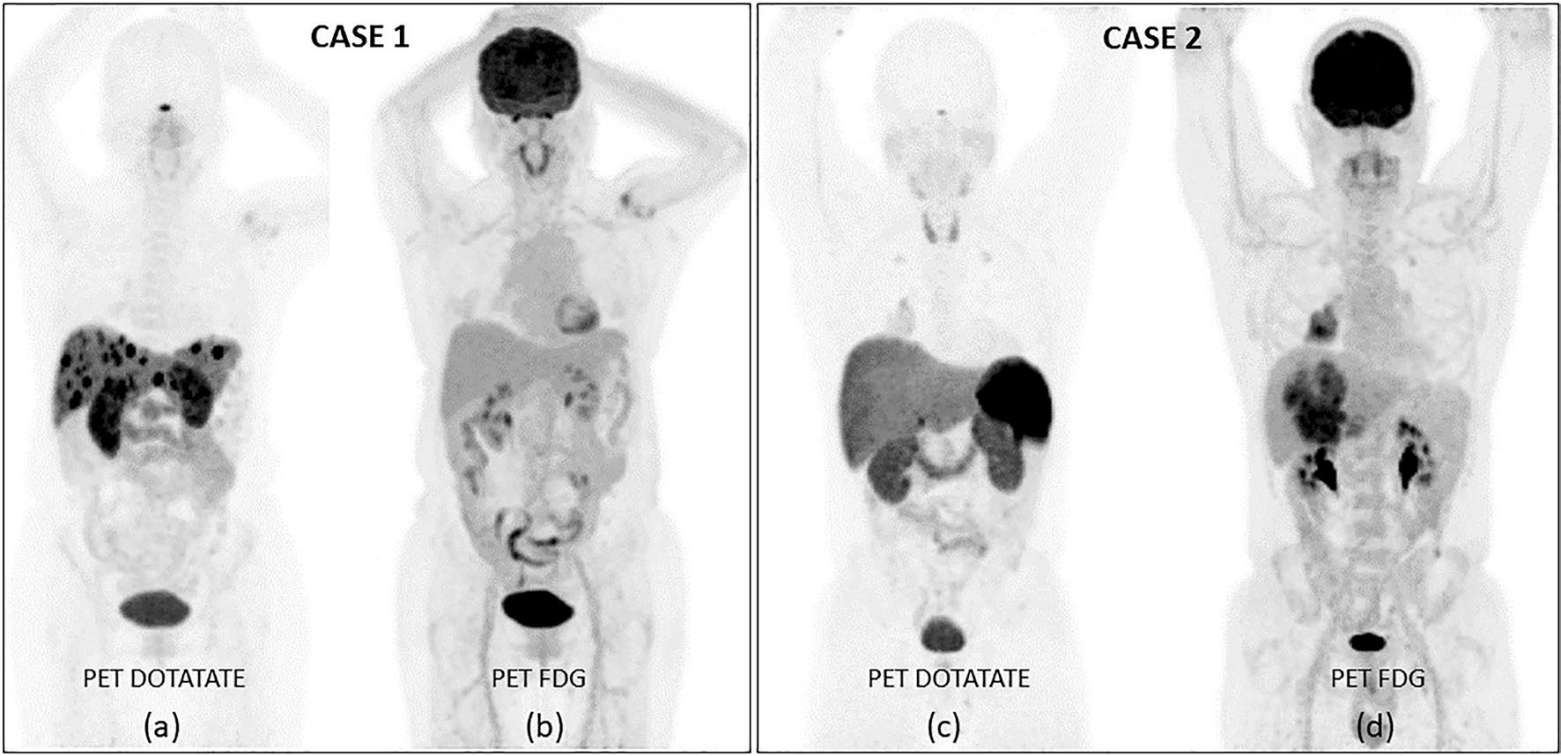
In case 1 (left), the **a** PET DOTATATE scan shows multiple hepatic SSTRI-positive lesions which have no uptake on the corresponding **b** PET FDG scan. The patient went on to successful treatment with LUTATE. In Case 2 (right), however, the **c** PET DOTATATE scan is essentially negative in the liver, while in **d** the PET FDG scan demonstrates significant uptake in both the lung lesion and the liver. This patient was deemed not suitable for treatment with LUTATE and was offered systemic medical therapy instead

Case 1 is a 63-year-old Asian female who underwent a distal pancreatectomy which showed a histological diagnosis of WHO Grade 2 pancreatic NET based on a Ki67 proliferative index of 16% and 16 mitoses per 10 high power fields (HPFs). A small number of liver metastases were demonstrated on the post-surgical DOTATATE PET scan 2 months later. In a follow-up DOTATATE scan 6 months later, there was evidence of rapid disease progression in the liver (Fig. 3a). An FDG PET scan at the time was normal with no evidence of any hepatic or other metabolically active disease (Fig. 3b). The subject was classified with a NETPET score of P1 and thus suitable for treatment with PRRT and subsequently received four cycles of ~ 8 GBq/cycle of LUTATE over 6 months, followed by reassessment with DOTATATE PET scanning 3 months after completion. Follow-up imaging demonstrated an excellent response.

By way of comparison, case 2 is a 50-year-old Caucasian male who was asymptomatic apart from a slight cough. An X-ray CT scan of the thorax revealed a well-defined mass in the lower lobe of the right lung plus a number of hepatic metastases. Subsequent biopsy confirmed these to be a well-differentiated neuroendocrine tumour of unknown origin, possibly atypical bronchial carcinoid, with Ki67 index of 15% and 12 mitoses per 10 HPFs. However, in this example the dual-PET imaging with DOTATATE and FDG PET scans revealed disease which was SSTRI negative, but strongly FDG positive (Fig. 3c, d). This patient was classified as NETPET score P5 and was deemed not suitable for PRRT due to the lack of uptake in any SSTR-expressing target and was instead offered combination capecitabine and temozolomide (“CAPTEM”) treatment.

## Discussion

The management and prognosis for patients with neuroendocrine tumours has changed dramatically in the past decade. The addition of widely available PRRT, a molecularly targeted systemic treatment, has dramatically changed the outlook for subjects with, in particular, grade 1 and 2 NETs. In Australia, PRRT is now available in all mainland capital cities and has recently been added in New Zealand as well. NETs are, by definition, heterogeneous in nature and origin. Nevertheless, if well differentiated and expressing abundant SSTRs, particularly subtypes SSTR_2_ and SSTR_5_, they can be treated with PRRT such as LUTATE if they demonstrate significant uptake on pre-treatment PET imaging. Even higher-grade (WHO grade 3) NETs are potentially treatable with PRRT if they demonstrate sufficient DOTATATE uptake on PET imaging. The theranostic paradigm involves demonstrating targetable tumour with pre-treatment imaging followed by PRRT with the same molecule, but with a therapeutic radionuclide substituted for the imaging radionuclide. Neuroendocrine carcinomas (NECs) usually exhibit low SSTR expression and should be treated with conventional chemotherapy.

The three main components of our approach to managing the NET patients are:

- establishing a NET multidisciplinary team at our centre,
- staffing it with NET specialists from the different disciplines and
- using morphological and functional molecular imaging to regularly restage the patient and guide treatment.

After 5 years of operating with this approach, the centre achieved certification as an ENETS *Centre of Excellence* in 2019. This is largely based on the MDT operating with defined protocols and adherence to guidelines at its regular meeting.

The results that have been presented in this report demonstrate the safety and efficacy of PRRT as a therapeutic option. We have followed European and North American Guidelines regarding the treatment protocols, with appropriate kidney protection to avoid renal tubular damage. Very few of our subjects demonstrated any renal toxicity. The conservative limit from external beam radiotherapy (EBRT) to the kidney is 23 Gy; however, due to the lower dose rate from the slowly decaying nuclei of ^177^Lu used in most PRRT, a limit of 30 or even 40 Gy is likely to be a more appropriate safe renal tolerance. In this audit, we found that we are delivering an average dose of around 10 Gy over the four cycles of LUTATE treatment. Likewise, bone marrow doses are extremely low and not likely to reach the accepted tolerance of 2 Gy in the standard four cycles of treatment with PRRT. This raises the question as to whether treatment could be more aggressive, by giving larger amounts of LUTATE during the four cycles, or whether the number of cycles could be readily extended. It would seem in most patients that 12 or more cycles could be tolerated without concern for off-target toxicities.

In this series of 142 subjects, we saw superior PFS and OS compared to the NETTER-1 trial, acknowledging the selection bias of a single-centre study versus a multicentre international study, and over and above the selection factors which we applied that NETTER1 did not, as described below. Our OS figures are almost 2 years greater than those from NETTER-1. We attribute much of this to the use of molecular imaging compared to what was available to participants in the earlier NETTER-1 study, many of whom did not have access to molecular PET imaging and had to rely on a very inferior SSTRI scan using indium-111. In our practice, we frequently combine the SSTRI of DOTATATE with a contemporaneous FDG PET scan. The FDG PET scan reflects increased glycolytic rate of cancer cells due to the Warburg effect (Warburg 1931). This allows us to perform a form of “whole body biopsy” where different clones of neuroendocrine tumour cells exhibiting different degrees of uptake of DOTATATE and FDG, reflecting varying degrees of dedifferentiation, can be assessed. When assessing the dual-PET imaging, we essentially look at two characteristics: the first is the degree of uptake in each of the two scans, and the second is the degree of spatial concordance in the abnormal foci. A high degree of SSTR uptake is generally regarded as a good feature, especially if there is little or no FDG uptake co-localised with it. High DOTATATE uptake also suggests good tumour targeting and radiation delivery from the PRRT. Conversely, low DOTATATE uptake is seen as a poor indicator of effective treatment with PRRT and this is compounded if there is significant FDG uptake in the same tissues, or there are areas of abnormal FDG uptake with little or no DOTATATE uptake. The latter cannot be targeted with PRRT. All of this is of course augmented by high quality morphological imaging using the appropriate modalities as required—CT and MRI with contrast and ultrasound.

When initially diagnosed, NETs are graded using histopathology from a surgical resection or biopsy. This grade continues with the patient until a subsequent biopsy or surgery demonstrates a change in grade. Our use of PET scanning acts as a surrogate for this regrading on tissue specimens, which is more invasive and can suffer from sampling issues. At the very least, dual-PET imaging can guide biopsy to sample different and perhaps more aggressive clones than the original disease.

## Conclusions

We have established a dedicated NET MDT to manage state-wide referrals from within NSW. During the period included in this audit around 420 individuals had been discussed by the NET MDT, while at the time of writing in 2022 over 900 individuals have been presented at the fortnightly MDT meeting since it commenced. The MDT includes NET-dedicated specialists from multiple disciplines. The service was recognised as an ENETS *Centre of Excellence* in 2019. Most patients receiving LUTATE tolerate the treatment extremely well with a measured incidence of myelodysplastic syndrome of 5%. In the first 5 years of treatments, we measured the median OS to be 72.5 months and median PFS of 32.3 months. The NET MDT relies on strong morphological and functional imaging with both PET DOTATATE and FDG to guide appropriate treatment selection. The NET MDT also provides a platform for recruiting suitable subjects to clinical trials (such as CONTROL NETS—ACTRN 12615000909527 (Pavlakis et al. 2022), which have the ability to generate high level clinical evidence. This evidence, in turn, feeds back into guidelines and clinical practice to give improved patient outcomes.

## Data Availability

The data used in this study represents Standard-of-Care clinical data and remains confidential to the Northern Sydney Local Health District.

## Footnotes

1 Theranostic: Thera(py) + (diag)nostic; the term “theranostic” is attributed to the American John Funkhouser in a press release from the company *Cardiovascular Diagnostics* in August 1998 (PharmaNetics and Aventis Pharmaceuticals announce filing of 510 K application for enoxaparin test [press release]. *J Nucl Med*. 2017;58(Supplement 2):3S-9S.).
